# Case report of recurrent epistaxis caused by a live leech in the nasal cavity

**DOI:** 10.1097/MD.0000000000037720

**Published:** 2024-04-05

**Authors:** Zi-Bin Yang, Yan Liu, Qiu-Juan Zhang, Bing-Ran Zhang, Ming-Wei Liu

**Affiliations:** a Department of Orthopedics, People’s Hospital of Dali Bai Autonomous Prefecture, Dali, Yunnan, China; b Department of Gastroenterology, The People’s Hospital of Lincang City, Lincang, Yunnan, China; c Department of Emergency, The First Affiliated Hospital of Kunming Medical University, Kunming, Yunnan, China; d Department of Emergency, People’s Hospital of Dali Bai Autonomous Prefecture, Dali, Yunnan, China.

**Keywords:** case report, diagnosis, live leech, nasal cavity bleeding, treatment

## Abstract

**Rationale:**

Epistaxis is one of the common emergencies in otolaryngology. There are many causes of epistaxis, but reports of epistaxis due to nasal foreign bodies like leeches are rare.

**Patient concerns:**

A 55-year-old male presented with “repeated epistaxis for over 20 days.” Nasal endoscopy revealed a live leech in the olfactory area of the left nostril.

**Diagnoses:**

The patient was diagnosed with epistaxis caused by a live leech in the nasal cavity.

**Interventions:**

Under nasal endoscopy, the leech was grasped with a vascular clamp and removed from the nasal cavity. The leech measured 8 cm in length. Hemostasis was achieved using a gelatin sponge at the wound site, and the nasal cavity was packed with Vaseline gauze.

**Outcomes:**

The live leech was removed via nasal endoscopy. Two days later, the Vaseline gauze packing was removed, and the patient experienced no further nasal bleeding.

**Conclusion:**

Live leeches in the nasal cavity can cause epistaxis. Nasal endoscopic removal of the live leech is an effective treatment.

**Lesson:**

There are many causes of epistaxis, which are nonspecific and prone to missed or incorrect diagnosis. In patients with a history of fieldwork or direct contact with leeches who present with recurrent nasal bleeding, the possibility of epistaxis caused by a live leech should be considered, and timely and effective treatment should be provided.

## 1. Introduction

Epistaxis, commonly encountered in otolaryngology, is an emergency with a high rate of self-resolution. Approximately 60% of people experience epistaxis at some point, and about 6% seek medical assistance.^[1,2]^ Among those requiring medical intervention, around 10% present with severe, potentially life-threatening cases.^[3]^ Particularly, recurrent intractable epistaxis often poses challenges due to its concealed bleeding sites and significant blood loss, making it difficult to detect and control.^[3,4]^ The etiology of epistaxis includes nasal disorders, local anatomical abnormalities, and certain systemic diseases. It can be triggered by both systemic and local factors.^[5]^ Local causes encompass nasal trauma, deviated nasal septum, nasal and nasopharyngeal tumors, nasal foreign bodies, rhinitis, sinusitis, etc, while systemic causes include cardiovascular diseases, acute febrile infectious diseases, nutritional disorders, vitamin deficiencies, poisoning, and hematological disorders. The nasal blood supply primarily derives from the anterior and posterior ethmoidal arteries and branches of the sphenopalatine artery from the maxillary artery. Epistaxis can occur at any age, with peak incidences observed in individuals below 10 years and those aged 70 to 79.^[6]^ However, epistaxis due to intranasal foreign bodies like leeches is rarely reported.

Leeches are categorized into terrestrial, aquatic, and parasitic types, with the former 2 being more commonly encountered.^[7]^ They typically inhabit freshwater and are prevalent in rice fields and rivers across China. Leeches feed on the blood of humans, livestock, and frogs. They attach to the skin to feed, secreting hirudin to prevent blood clotting, causing local anesthesia, vasodilation, persistent bleeding, and edematous skin rashes with slight pain.^[8]^ Leeches can also enter body orifices such as the nasal cavity, mouth, anus, vagina, and urethra, causing itching or bleeding in these areas, and can reside for several years without detachment.^[9]^ Epistaxis caused by leeches lacks specificity and is easily misdiagnosed or overlooked clinically. This article reports on the diagnosis and treatment of recurrent epistaxis caused by a live leech in the nasal cavity, aiming to guide clinicians in considering leech-induced epistaxis, especially in patients with outdoor activities, to provide timely and effective treatment.

## 2. Case report

### 2.1. Medical history

The patient, a 54-year-old male, experienced an incident 3 weeks prior where he inadvertently sucked a leech into his mouth while trying to unblock a water pipe at home. He immediately spat it out. Following this, he suffered from recurrent nasal bleeding for 3 weeks, varying in intensity. He had previously sought treatment at other hospitals where he was treated for bleeding due to nasal mucosal erosion, but his condition did not improve. He presented to the First Affiliated Hospital of Kunming Medical University for treatment on November 30, 2023.

### 2.2. Past medical history

The patient denied any history of hypertension, diabetes, cardiovascular, pulmonary, hematological, endocrine diseases, or any infectious diseases. He also had no history of trauma, surgery, blood transfusion, or allergies. Vaccination history was unclear.

### 2.3. Physical examination

The patient’s temperature was 36.3°C, pulse rate was 125 beats/minute, respiratory rate was 18 breaths/minute, and blood pressure was 143/90 mm Hg. His general condition was fair, with no obvious signs of anemia. No enlargement of superficial lymph nodes throughout the body. The skull and face appeared normal, with both eyeballs presenting normally. No enlargement of the thyroid gland. The chest wall was normal, with coarse breath sounds in both lungs and no auscultation of dry or moist rales. The heart borders were not enlarged, with a heart rate of 125 beats/minute and a regular rhythm. No pathological murmurs were heard in any valve areas. The abdomen was soft with no tenderness or rebound pain. The liver was not palpable, and bowel sounds were normal.

### 2.4. Laboratory data

On November 30, 2023, the complete blood count showed a white blood cell count of 5.72 × 10^9^/L, neutrophils 74.3%, lymphocytes 8.1%, red blood cells 5.71 × 10^12^/L, hemoglobin 162 g/L, and platelets 328 × 10^9^/L. Coagulation and fibrinolysis system tests revealed a prothrombin time of 11.9 seconds, prothrombin activity of 126%, international normalized ratio of 1.02, prothrombin time ratio of 0.93, reagent sensitivity index of 1.29, fibrinogen of 4.57 g/L, clotting time of 18.6 seconds, activated partial thromboplastin time of 43.1 seconds; fibrin degradation products at 1.6 mg/L, D-dimer at 0.42 mg/L, and antithrombin III at 85%. Liver and kidney functions, electrolytes, and cardiac enzymes were all within normal limits.

### 2.5. Nasal endoscopy

**Figure 1.**
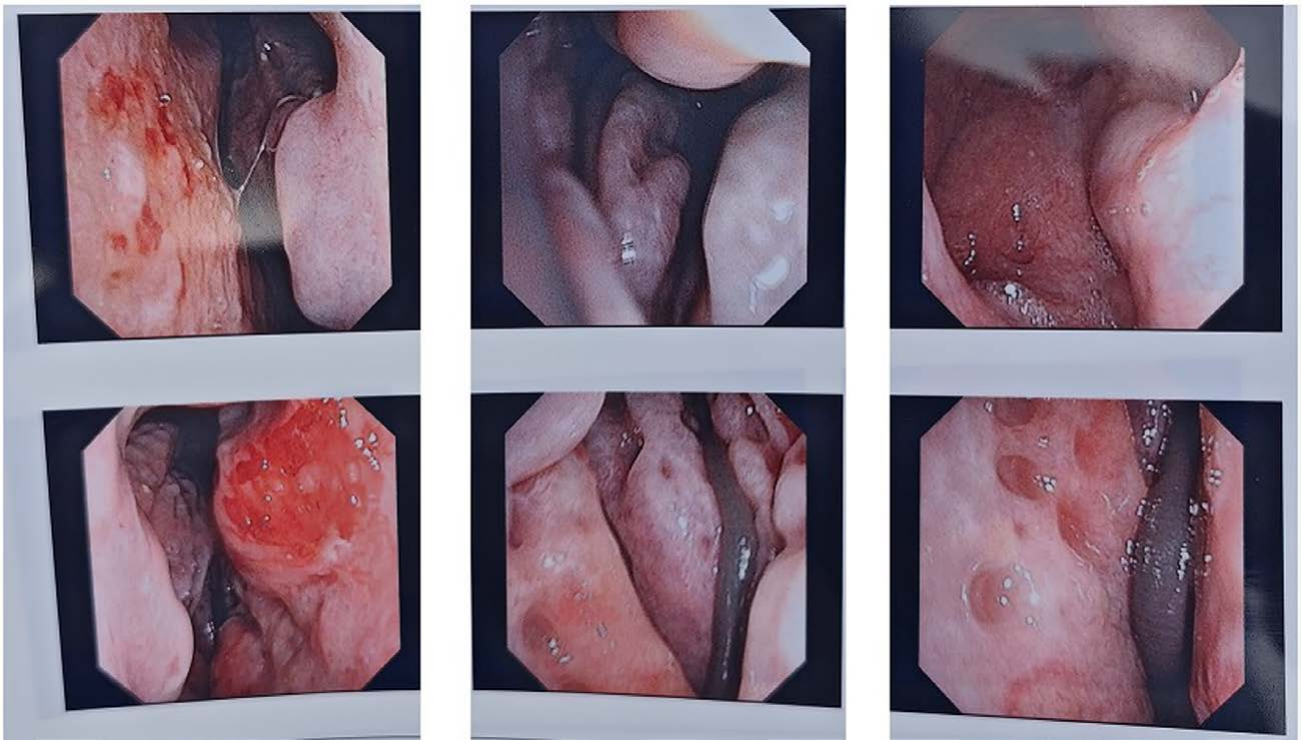
Endoscopic view of the patient’s nasopharyngeal area.

Nasal endoscopy revealed erosion and ulceration in both sides of the nasal septum’s Little’s area, pale nasal mucosa, sanguinous changes in the inferior turbinates, and a live foreign body (leech) visible in the left olfactory area (Fig. 1). The nasopharynx appeared smooth, with both sides of the Eustachian tube’s pharyngeal orifice and pharyngeal recess smooth and intact (Fig. 1).

### 2.6. Diagnosis and treatment

Based on the medical history and the nasal endoscopy findings of a leech, as well as nasopharyngeal lesions, the patient was diagnosed with nasal bleeding caused by a live leech in the nasal cavity. Under direct vision with an anterior rhinoscope, the leech was gently, accurately, and swiftly clamped at its attachment site and pulled out. Hemostasis at the wound site was achieved with a gelatin sponge, and the nasal cavity was packed with Vaseline gauze. The live leech was removed, and 2 days later, the Vaseline gauze packing was also removed.

### 2.7. Follow-up after treatment

One week after discharge, the patient had no recurrence of nasal bleeding. He was advised to seek immediate medical treatment if he experienced recurrent nasal bleeding after fieldwork or direct contact with leeches, as live leeches can cause epistaxis.

## 3. Discussion

Leeches parasitize the nasal cavity to feed on blood, secreting hirudin during feeding, which causes epistaxis and recurrent episodes.^[10,11]^ Prolonged infestation can lead to hemorrhagic anemia and is often misdiagnosed as other nasal bleeding disorders. The movement of the leech can cause peculiar itching and discomfort inside the nose, leading to misdiagnosis as allergic rhinitis. Leeches, being photophobic, often reside in darker areas of the nasal cavity such as the nasal apex, olfactory fissure, and posterior part of the nasal cavity, making them hard to detect during routine anterior rhinoscopy examinations. In this case, the patient’s recurrent epistaxis was initially misdiagnosed as bleeding due to nasal mucosal erosion at another hospital, with no improvement following treatment.

### 3.1. Key diagnostic points

Recurrent intermittent epistaxis in patients engaged in rural labor; History of drinking water from streams or consuming unclean water prior to the onset of symptoms; Symptoms may include nasal itching, a foreign body sensation, nasal congestion, sneezing, etc; In some cases, patients have reported seeing a “leech extending out of the nostril”; This type of nasal bleeding is unresponsive to medicinal treatment; Examination of the nasal cavity may reveal a brown or tan-striped mass that retracts and moves upon touch. In this patient’s case, the day before symptom onset, he accidentally sucked a leech into his mouth while trying to unblock a water pipe and then spat it out. Subsequently, he experienced recurrent nasal bleeding. Therefore, the history of consuming unclean water and contact with a leech, followed by recurrent nasal bleeding, strongly suggested the possibility of a live leech in the nasal cavity causing the bleeding. Ultimately, this diagnosis was confirmed through nasal endoscopy.

### 3.2. Treatment points

The preferred method is to use forceps without anesthesia, requiring the actions to be “gentle, precise, steady, firm, and slow.” Successfully removing the leech in one go is crucial, as the body of the leech is tough and highly elastic, capable of withstanding significant pulling force. The appropriate pulling force and duration of effort are key to successful surgery. Since leeches have strong suction, excessive and rapid pulling during clamping can cause the body of the leech to break or slip, making removal difficult and potentially causing tears in the nasal mucosa. As soon as the forceps touch the leech, they should quickly and firmly clamp down without loosening, then slowly and steadily pull outward. Continuing this for about 3 to 5 seconds is usually enough to completely remove the leech. Ensure no residual leeches are left in the nasal cavity for the surgery to be considered successful. Generally, only 1 leech is found in patients, ranging in length from 2 to 8 cm^[12]^; If the initial attempt with forceps is unsuccessful and the leech retracts, making direct removal challenging, or if nasal cavity anatomical variations make direct removal difficult, then 2% lidocaine should be used for topical anesthesia before another attempt. When anesthetized, the leech becomes immobile or moves slowly, making it easier to remove; If the leech moves deeper into the nasal cavity and direct removal with forceps is challenging, a nasal endoscope may be needed to locate and remove it. In this case, we successfully removed a leech approximately 8 cm long using a nasal endoscope.

## 4. Strengths and limitations

### 4.1. Strengths

This case report demonstrates that live leeches in the nasal cavity can cause recurrent epistaxis and that removal under nasal endoscopy is an effective treatment.

### 4.2. Limitations

Symptoms of nasal bleeding caused by live leeches in the nasal cavity are nonspecific, leading to potential misdiagnosis or missed diagnosis. For patients with a history of fieldwork or direct contact with leeches who present with recurrent nasal bleeding, the possibility of epistaxis caused by a live leech should be considered, and timely and effective treatment should be provided.

## 5. Conclusion

Recurrent epistaxis caused by live leeches in the nasal cavity is not specific, leading to frequent misdiagnoses or missed diagnoses. Therefore, a detailed medical history is crucial for patients with recurrent epistaxis, especially those from rural areas who experience a sensation of worms crawling in their nose. Inquiries should not only be made about drinking unfiltered water or swimming history but also about the hygiene of household water sources. For patients with a history of fieldwork or direct contact with leeches who present with recurrent nasal bleeding, the possibility of epistaxis caused by a live leech should be considered, and timely and effective treatment should be provided. Additionally, leeches exhibit photophobia and retract deeper into the nasal cavity when exposed to bright light. Therefore, for patients suspected of having a leech in the nasal cavity, vascular forceps should be prepared in advance to prevent prolonged attempts at extraction. Leeches possess suckers and tend to tightly adhere to the nasal mucosa when in pain, so care must be taken not to apply excessive force during removal to avoid tearing the nasal mucosa or breaking the leech. Furthermore, leeches have strong regenerative abilities. If not completely removed, the residual part can grow into a complete leech and continue to survive, leading to treatment failure.

## Acknowledgments

This work was supported by the Nature Science Foundation of China under Grant (NO. 81960350); Yunnan Applied Basic Research Project-Union Foundation of China under Grant (NO. 202201AY070001-091).

## Author contributions

**Conceptualization:** Zi-Bin Yang, Yan Liu, Ming-Wei Liu.

**Data curation:** Zi-Bin Yang, Yan Liu, Qiu-Juan Zhang, Bing-Ran Zhang.

**Formal analysis:** Qiu-Juan Zhang, Ming-Wei Liu.

**Funding acquisition:** Ming-Wei Liu.

**Investigation:** Zi-Bin Yang, Yan Liu, Ming-Wei Liu.

**Methodology:** Qiu-Juan Zhang, Bing-Ran Zhang, Ming-Wei Liu.

**Project administration:** Zi-Bin Yang, Yan Liu, Qiu-Juan Zhang, Ming-Wei Liu.

**Resources:** Bing-Ran Zhang.

**Software:** Zi-Bin Yang, Yan Liu, Bing-Ran Zhang, Ming-Wei Liu.

**Supervision:** Zi-Bin Yang, Yan Liu, Qiu-Juan Zhang, Ming-Wei Liu.

**Validation:** Zi-Bin Yang, Bing-Ran Zhang.

**Visualization:** Yan Liu, Qiu-Juan Zhang, Bing-Ran Zhang, Ming-Wei Liu.

**Writing – original draft:** Qiu-Juan Zhang, Ming-Wei Liu.

**Writing – review & editing:** Zi-Bin Yang, Qiu-Juan Zhang, Ming-Wei Liu.
